# First-principles investigation of the ferroelectric, piezoelectric and nonlinear optical properties of LiNbO_3_-type ZnTiO_3_

**DOI:** 10.1038/s41598-019-53986-6

**Published:** 2019-11-26

**Authors:** Jing Zhang, Bin Xu, Yu-Sheng Wang, Zhen Qin, San-Huang Ke

**Affiliations:** 1 School of of Physics and Electronics, North China University of Water Resources and Electric Power, ZhengZhou, 450045 China; 2 School of Physics Science and Engineering, Tongji University, 1239 Siping Road, Shanghai, 200092 P.R. China

**Keywords:** Nonlinear optics, Electronic properties and materials

## Abstract

The newly synthesized LN-type ZnTiO_3_ (J. Am. Chem. Soc. 2014, 136, 2748) contains cations with the electronic configurations nd^10^ (Zn^2+^: 3d^10^) along with second-order Jahn-Teller (SOJT) nd^0^ (Ti^4+^: 3d^0^) cations. This is different from traditional ferroelectrics with the electric configurations of d^0^ transition metal ions or/and lone pair electrons of ns^2^. Using a first-principles approach based on density functional theory, we investigate the electronic structure, zone-center phonon modes, piezoelectric and nonlinear optical properties of the LiNbO_3_-type ZnTiO_3_. The electronic structure indicates that this compound is a wide direct-band-gap insulator. The results reveal that this compound is a good ferroelectric material with a large spontaneous polarization of 90.43μC/cm^2^. The Raman scattering peaks of A_1_ and E modes are assigned to their zone-center optical modes. Additionally, the large piezoelectric and nonlinear optical susceptibilities reveal that LiNbO_3_-type ZnTiO_3_ is a high-performance lead-free piezoelectric and nonlinear optical crystal.

## Introduction

In recent years, noncentrosymmetric (NCS) compounds have attracted great interest in physics and material science because of their symmetry-dependent ferroelectric, piezoelectric and second-order nonlinear optical (NLO) properties, etc^1,2^. When searching for other polar oxides, researchers pay special attention to two classes of compounds, specifically those that include second-order Jahn-Teller distorted (SOJT) cations (Te^4+^, Sn^4+^ Ti^4+^, Mo^6+^, Nb^5+^, V^5+^, etc.) and cations with stereoactive lone pair electrons of ns^2^ (Bi^3+^, Pb^2+^, Se^4+^, etc.)^3–12^. For the NCS compounds, there are two famous structures: perovskite-type (Pv-type) and LiNbO_3_-type (LN-type) phases. Taking into account the structural relationship between the above two, the LiNbO_3_-type structure can be regarded as the derivative of the perovskite structure^13^. In general, most of the LN-type compounds cannot be synthesized under general conditions and instead require high temperature and high pressure.

**Figure 1:**
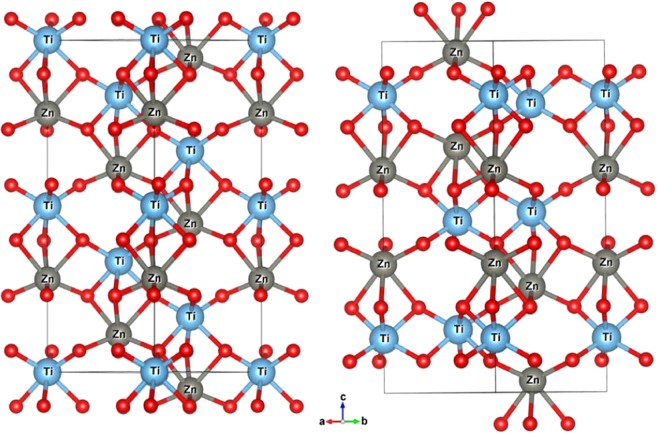
The crystallographic structures of LiNbO_3_ (LN)-type ZnTiO_3_ (left, space group R3c) and ilmenite (IL)-type ZnTiO_3_ (right, space group $R\bar{3}$) along the z-axis.

**Figure 2:**
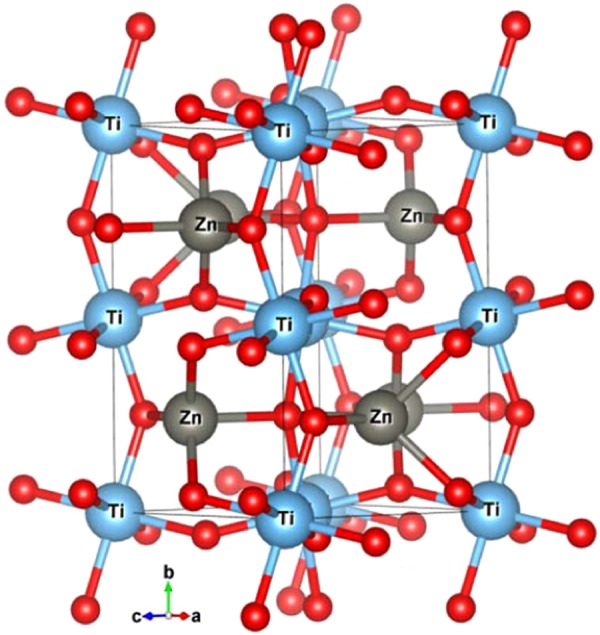
A view of the crystal structure of perovskite(Pv)-type ZnTiO_3_ (space group Pnma) along the y-axis.

In 2014 a new NCS compound, the LN-type ZnTiO_3_, was synthesized by Inaguma *et al*. under high temperature and high pressure^14^. Their results show that LN-type ZnTiO_3_ has a large spontaneous polarization and greater second harmonic generation (SHG) response compared to LiNbO_3_ and LN-type ZnSnO_3_. According to Bartram *et al*.^15^, Ito *et al*.^16^ and Inaguma *et al*.’s reports^14^, two paraelectric phases of ZnTiO_3_ are ilmenite (IL)-type (hexagonal space group $R\bar{3}$) and Pv-type (orthorhombic space group Pnma) structures, respectively. Ito *et al*. reported that IL-type ZnTiO_3_ decomposes into ZnO and TiO_2_ under a pressure of 20∼25 GPa^16^; inspired by this, Inaguma *et al*. successfully synthesized LN-type ZnTiO_3_ at less than 20 Gpa. As discussed by Navrotsky^13^ and Linton *et al*.^17^, the LN-type compounds such as ZnSnO_3,_ FeTiO_3_, MnTiO_3_ and ZnGeO_3_, etc. are metastable and quenched products from the high-pressure perovskite phases^18–24^. This means that LN-type ZnTiO_3_ is regarded as a retrograde product from the high-pressure perovskite phase on pressure release^14^. The crystal structures of ZnTiO_3_ in the ferroelectric and two paraelectric phases are presented in Figs. 1 and 2, respectively.

Notice that recently-synthesized LN-type ZnSnO_3_ and CdPbO_3_ are novel polar oxides (hexagonal space group R3c) that include only the cations with the electronic configurations of nd^10^ (Zn^2+^ 3d^10^, Cd^2+^ 4d^10^, Sn^4+^ 4d^10^, Pb^4+^ 5d^10^) and provide a new strategy for synthesizing new polar compounds. The LN-type ZnTiO_3_ contains not only Zn^2+^ (3d^10^), but also Ti^4+^ (3d^0^), the former and later belonging to nd^10^ and SOJT nd^0^ cations, respectively. Although LN-type ZnTiO_3_ and ZnSnO_3_ have the similar crystal structures, experimental results have shown that the SHG intensity of LN-type ZnTiO_3_ is 24 times greater than that of LN-type ZnSnO_3_^14^. The space group of LN-type ZnTiO_3_ belongs to the 3 m polar point group, so this compound is a promising candidate as a piezoelectric and nonlinear optical material. Therefore, LN-type ZnTiO_3_ provides a perfect example for comparing the influence that the nd^10^ and SOJT nd^0^ cations have in producing the ferroelectric, piezoelectric and nonlinear optical behavior.

Nowadays, first-principles calculations based on density functional theory (DFT) play an important role in meeting the needs of experimenters because they can accurately predict the properties of new materials at the atomic scale. In general, the measurements of piezoelectric and nonlinear optical properties require very high crystal quality, specialized laboratories, and are expensive, so first-principles calculations provide an alternative to predicting optical and piezoelectric properties, and can be microscopically explain the relationship between structure and properties. First-principles calculations including the modern theory of polarization^25,26^, linear corresponding approaches, as well as the density functional perturbation theory (DFPT)^27–29^, can help us study the ferroelectric, piezoelectric, and nonlinear optical properties of materials. In addition to traditional ferroelectrics, first-principles calculations have made great progress in predicting and investigating new nonlinear optical crystal^30,31^, multiferroic materials^32,33^, two-dimensional ferroelectrics^34^ and ferroelectric metal^35,36^, etc. In order to study the intrinsic correlation from the paraelectric to ferroelectric phase transition, we have investigated the zone-center phonon modes of the ilmenite, perovskite and LiNbO_3_-type ZnTiO_3_ phases, respectively. Additionally, there have been no prior reports on the piezoelectric and nonlinear optical properties, including nonlinear optical susceptibilities and electro-optical coefficients, of LN-type ZnTiO_3_, and so in this paper we carry out first-principles calculations to investigate the origin of the ferroelectric behavior, and analyze the piezoelectric and nonlinear optical properties of LN-type ZnTiO_3_ for the first time. Since the piezoelectric and nonlinear optical properties of this type of novel material with nd^10^ and SOJT nd^0^ cations have not been investigated previously, our calculations provide an important complement to experimental research.

## Method

Our calculations are based on the framework of density functional theory (DFT). We mainly used the ABINIT package^37,38^. In order to calculate the electronic structure, spontaneous polarization, zone-center phonon modes, piezoelectric and nonlinear optical properties, we adopt norm-conserving pseudopotentials based on the local density approximation (LDA) as exchange-correlation potentials. An 8 × 8 × 8 Monkhorst-Pack k-point mesh is used with a plane wave cut-off of 45 hartrees. The Zn 3d, 4s and Ti 3d, 4s, as well as O 2s, 2p electrons are regarded as valence states to construct the pseudopotentials. The electronic structures are also calculated by adopting ultrasoft pseudopotentials^39^ based on the Vienna ab initio simulation package (VASP)^40–42^, and the exchange-correlation potentials are based on LDA and the general gradient approximation (GGA) with Perdew-Burke-Ernzerhof (PBE) functional^43,44^, respectively. Additionally, due to DFT systematically underestimate band gaps with respect to experiment, the Heyd-Scuseria-Ernzerhof (HSE) screened hybrid functional (with the version HSE06)^45,46^ is used to calculated the band structures.

The calculations of the polarization and piezoelectric properties are based on linear response functions, while the computations of nonlinear optical properties are based on nonlinear response functions. The linear and nonlinear response calculations are performed using density functional perturbation theory (DFPT). In order to calculate the above physical properties, the homogeneous electric fields, atomic displacements and stresses are regarded as perturbations. When stress is applied to a piezoelectric crystal, its interior will produce a polarization phenomenon where opposing positive and negative charges appear on its two opposite surfaces. This phenomenon is called the positive piezoelectric effect. On the contrary, when the electric field is applied in the polarization direction of the piezoelectric crystal, it will also deform. After the electric field is removed, the deformation of the piezoelectric crystal disappears. This phenomenon is called the reverse piezoelectric effect. In this work, we calculate the piezoelectric stress tensor from the zero-field derivative of polarization by using stress as a perturbation:

1 $${e}_{\alpha i}={\frac{\partial {p}_{\alpha }}{\partial {\eta }_{i}}|}_{E}$$

where η and i represent second-rank stress tensor and the Cartesian coordinate direction. In the actual computation, the piezoelectric stress tensor comes from the response from fixed ions and relative displacement of the ions:

2 $${e}_{\alpha i}={\frac{\partial {p}_{\alpha }}{\partial {\eta }_{i}}|}_{\mu }+\sum _{k}\frac{\partial {p}_{\alpha }}{\partial {\mu }_{\alpha }(k)}\frac{\partial {\mu }_{\alpha }(k)}{\partial {\eta }_{i}}$$

where μ represents the displacement of the ions. For an insulator, the relation between the polarization and macroscopic electric field can be described as follows:

3 $${P}_{i}={P}_{i}^{s}+\mathop{\sum }\limits_{j=1}^{3}{x}_{ij}^{(1)}{\varepsilon }_{j}+\mathop{\sum }\limits_{j,l=1}^{3}{x}_{ijl}^{(2)}{\varepsilon }_{j}{\varepsilon }_{l}+\ldots ,$$

where ${P}_{i}^{s}$ represents spontaneous polarization in zero electric field; ${x}_{ij}^{(1)}$ are linear dielectric constants, and ${x}_{ijl}^{(2)}$ are second-order nonlinear optical (NLO) coefficients. The NLO and electro-optic (EO) coefficients can be calculated from third-order energy derivatives within the 2n + 1 theorem^47^. Like LiNbO_3_ and LN-type ZnSnO_3_, we use the more convenient d tensor to represent NLO coefficients ${x}_{ijl}^{(2)}({d}_{ijl}=\frac{1}{2}{x}_{ijl}^{(2)})$. When applying an electric field to some crystals, especially piezoelectric crystals, their refractive index changes. This characteristic of a crystal can be described by refractive index ellipsoids. The phenomenon is known as the linear electro-optic (EO) effect or Pockels Effect:

4 $$\Delta {(\frac{1}{{n}^{2}})}_{i}=\mathop{\sum }\limits_{j=1}^{3}{r}_{ij}{\varepsilon }_{j}$$

where n and r_ij_ represent refractive index and linear EO coefficients (i = 1~6, and j = 1~3), respectively. Pockels effect exists only in the NCS crystals. The electro-optical (EO) tensor arises from the sum of three contributions: the electronic part, ionic contribution and piezoelectric effects^48,49^.

## Results and Discussions

### Electronic structure

**Figure 3:**
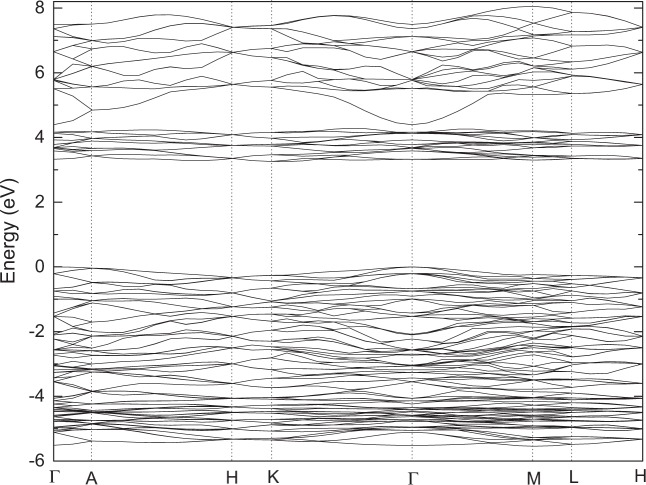
The band structures of LiNbO_3_-type ZnTiO_3_ along the high symmetry directions in the Brillouin zone based on norm-conserving pseudopotentials within GGA.

**Figure 4:**
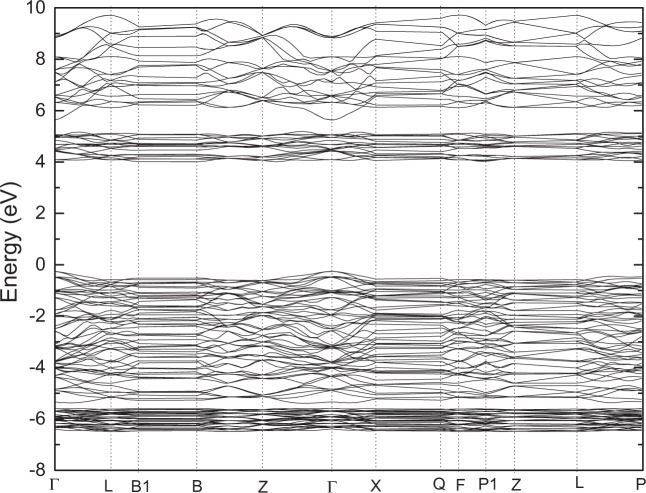
The band structures of LiNbO_3_-type ZnTiO_3_ along the high symmetry directions in the Brillouin zone based on HSE06.

Firstly, we perform the full geometry optimization to determine the crystal parameters in the paraelectric and ferroelectric phases. According to the optimized geometry (see Table 1), we have performed the electronic structure calculations of the LiNbO_3_-type ZnTiO_3_ based on ultrasoft and norm-conserving pseudopotentials and HSE06, respectively. Figures 3 and 4 plots the energy band structure of this compound along the high symmetry points in the first Brillouin zone based on norm-conserving pseudopotentials within GGA and HSE06. When calculating the energy band based on HSE06, more high symmetry points in the first Brillouin zone are used. From Fig. 3, it is found that the top of the valence band (VB) and the bottom of the conduction band (CB) are both located at Γ(0.0, 0.0, 0.0), so this compound is a direct band gap insulator. The calculated energy gaps (e_g_) are as follows: 3.054 eV (LDA) and 3.252 eV (GGA) based on norm-conserving pseudopotentials; 2.860 eV (LDA) and 2.956 eV (GGA) based on ultrasoft pseudopotentials. Therefore, the energy gap of norm-conserving pseudopotentials is slightly larger than that of ultrasoft pseudopotentials. From Fig. 4, it is found that the e_g_ based on HSE06 (see in Fig. 4) are 4.25 eV. So, the band gap based on HSE06 is the largest and is about 1 eV higher than that of GGA based on norm-conserving pseudopotentials. From Table 1, one can see that the lattice parameters of LDA and GGA are slightly lower and higher than the experimental values. The difference in the band gaps between GGA and LDA result from the difference in calculated lattice parameters. Compared with the results of LDA and GGA, the lattice parameters based on HSE06 are the lowest, however the band gap is the largest. Until now,as far as we know there is no experimental report on the band gap of LN-type ZnTiO_3_. Although the band gap of HSE06 is relatively larger than that of LDA and GGA, the agreement with the experimental band gap needs further verification.

**Table 1:** The calculated Lattice constants and volumes of the ilmenite (IL)-type (space group $R\bar{3}$) and Perovskite (Pv)-type (space group Pnma) and LiNbO_3_-type (space group R3c) ZnTiO_3_ based on norm-conserving (NC), ultrasoft pseudopotentials and Heyd-Scuseria-Ernzerhof screened hybrid functional (HSE06).

ZnTiO_3_ ($R\bar{3}$)	a (Ǻ)	b (Ǻ)	c (Ǻ)	V (Ǻ^3^)
Expt ref. ^15^	5.079	5.079	13.9270	311.1319
Present (LDA-NC)	5.0564	5.0564	13.9001	307.7801
Present (GGA-NC)	5.1647	5.1647	14.3519	331.5384
Present(GGA-PBEsol-NC)	5.1169	5.1169	14.1530	320.9172
**ZnTiO**_**3**_ **(Pnma)**	**a (Ǻ)**	**b (Ǻ)**	**c (Ǻ)**	**V (Ǻ**^**3**^**)**
Present (LDA-NC)	5.2026	7.4146	5.0631	195.3118
Present (GGA-NC)	5.3258	7.5722	5.18200	208.9797
Present(GGA-PBEsol-NC)	5.2751	7.4955	5.131523	202.8997
**ZnTiO**_**3**_ **(R3c)**	**a (Ǻ)**	**b (Ǻ)**	**c (Ǻ)**	**V (Ǻ**^**3**^**)**
Expt ref. ^14^	5.09452(12)	5.09452(12)	13.7177(3)	308.332(12)
Present (GGA-NC)	5.2089	5.2089	13.9559	327.9307
Present (LDA-NC)	5.0878	5.0878	13.5610	304.0071
Present(GGA-PBEsol-NC)	5.1579	5.1579	13.7210	316.1275
Present (GGA- ultrasoft)	5.1384	5.1384	13.9478	318.9250
Present (LDA- ultrasoft)	5.0211	5.0211	13.5725	296.3407
Present (HSE06)	5.0678	5.0678	13.6395	303.3668

**Figure 5:**
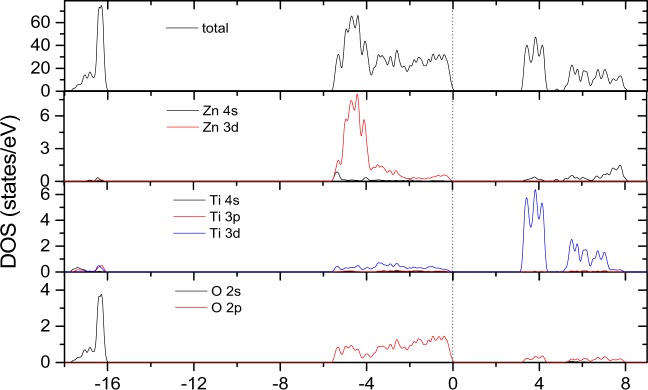
Total and partial density of states (DOSs) of LN-type ZnTiO_3_ based on norm-conserving pseudopotentials within GGA with the Fermi levels at 0 eV.

Figure 5 shows the total and partial density of states (DOSs) of LN-type ZnTiO_3_ based on norm-conserving pseudopotentials within GGA. According to this figure, in the energy region from −18 to −16 eV, the total DOS mainly arises from O 2p electrons, with a small contribution from Zn 4s electrons and Ti 3p, 3d and 4s electrons. In the energy range from −6 to −0 eV, the total DOS mainly arises from Zn 3d, Ti 3d and O 2s electrons, and there are obviously hybridizations between Zn 3d-O 2p and Ti 3d-O 2p states. Above the Fermi level, in the range 3 to 8 eV, the total DOS mainly arises from Ti 3d, Zn 3d and O 2p electrons. The calculated Mulliken charge populations of this compound are as follows: for Zn atom, the charge populations of the s, p and d orbitals are 0.27, 0.70 and 9.98; for Ti atom, the charge population of the s, p and d orbitals are 2.37, 6.43 and 2.15; for O atom, the charge populations of the s and p orbitals are 1.85 and 4.85. In addition, the calculated charge populations between nearest-neighboring (1.8652 Å) and next nearest-neighboring (2.1589 Å) Ti-O bonds are 0.56 and 0.30; the charge populations between the nearest-neighboring (2.0421 Å) and next nearest-neighboring (2.2671 Å) Zn-O bonds are only 0.29 and 0.04. The number of charges on the different bonds can reflect the covalent and ionic properties. Obviously, the chemical bonding of the Ti-O and nearest-neighboring Zn-O bonds are of mixed covalent-ionic character, however the next nearest-neighboring Zn-O bonds show ionic character. As discussed above, the hybridization of Zn-O bonds and Ti-O bonds mainly occurs in their 3d and 2p orbitals. The charge distribution of Zn-O and Ti-O bonds indicates that the empty 3d orbital in Ti^4+^ (3d^0^) more easily forms a covalent bond with O atoms than the fully occupied 3d orbital in Zn^2+^ (3d^10^). This may be the reason why the piezoelectric and nonlinear optical properties of LN-type ZnTiO_3_ are superior to those of the LN-type ZnSnO_3_ and LN-type ZnGeO_3_ (see the results below). In the latter two compounds, it is noted that the all the cations are fully occupied 3d orbitals.

### Spontaneous polarization

The most basic characteristics of ferroelectric materials is the existence of spontaneous polarization over certain temperature ranges, and the fact that the polarization can be reversed by the external electric field. Using the Berry-phase approach proposed by R. D. King-Smith and D. Vanderbilt^25,26^, we have calculated the spontaneous electric polarization of LN-type ZnTiO_3_ based on finite electric field calculations. The results show that the polarizations of P_x_ and P_y_ are almost equal to zero, so the polarization lies mainly along the z-axis direction. The total polarization is composed of the ionic (P_ion_) and electronic (P_ele_) contributions. The results based on LDA show that the total polarization is 90.43 μC/cm^2^, and the values of P_ion_ and P_ele_ are 89.07 μC/cm^2^ and 1.36 μC/cm^2^, respectively. Therefore, the electric polarization mainly arises from the ionic contribution. Based on the result of our GGA calculation, the total polarization is 93.14 μC/cm^2^, the vales of P_ion_ and electronic P_ele_ are 91.73 μC/cm^2^ and 1.41 μC/cm^2^. According to ref. ^14^, the values of polarization obtained by Born effective charges and nominal charge are 88 μC/cm^2^ and 75 μC/cm^2^. Our results are slightly larger than their values and consistent with their results based on Born effective charges.

The Born effective charges of the LN-type ZnTiO_3_ are presented in Table 2. The nominal charges of Zn, Ti and O are +2, +4 and −2, respectively. By comparing the Born effective charges of these ions and their nominal values, it is found that charge numbers Z^*^ of Zn, Ti and O (especially for Z_xx_^*^ and Z_yy_^*^ in Ti atom, Z_yy_^*^ in O1, and Z_xx_^*^ in O2 atoms) are obvious anomalous, which is an important characteristic in ferroelectric materials. From Table 2, one can see that Z^*^ of Zn and Ti atoms mainly occur on the diagonal elements, and the values of the non-diagonal elements are small or equal to zero. This is in consistent with the spontaneous polarization being mainly along the z-axis direction. Compared with Z^*^ of Zn and Ti atoms, the Z^*^ tensor of O atoms displays strong anisotropy, and this should be attributed to the structural distortions caused by the Zn 3d-O 2p and Ti 3d-O 2p orbital hybridizations.

**Table 2:** The calculate Born effective charge of Zn, Ti, O_1_∼_3_ in LN-type ZnTiO_3_.

Atom	Z_xx_^*^	Z_yy_^*^	Z_zz_^*^	Z_xy_^*^	Z_yx_^*^	Z_xz_^*^	Z_zx_^*^	Z_yz_^*^	Z_zy_^*^
Zn	2.59	2.59	2.29	0.24	−0.24	0.00	0.00	0.00	0.00
Ti	6.16	6.16	5.34	−0.54	0.54	0.00	0.00	0.00	0.00
O_1_	−2.19	−3.64	−2.54	−0.58	−0.55	−0.31	−0.35	−1.28	−1.32
O_2_	−3.77	−2.06	−2.54	−0.35	−0.34	1.27	1.32	0.37	0.37
O_3_	−2.79	−3.04	−2.54	0.90	0.92	−0.96	−0.97	0.91	0.95

### Zone-center phonon modes

In ferroelectric crystals that have undergone the paraelectric to ferroelectric transition, the relative displacements of anions and cations will lower the symmetry and lead to spontaneous polarization. This phenomenon can be explained by the theory of soft zone-center phonon modes. Firstly, we perform the symmetry analyses in ZnTiO_3_ and divide its zone-center phonon modes into irreducible representations. The two paraelectric phases of ZnTiO_3_ are ilmenite (IL)-type (hexagonal space group $R\bar{3}$) and Perovskite (Pv)-type (orthorhombic space group Pnma) structures, and the ferroelectric phase is LiNbO_3_-type structure (hexagonal space group R3c). In this compound, there exists a continuous phase transition from ilmenite-type structure to perovskite-type, and then to LiNbO_3_-type structure.

In high-symmetry IL-type ZnTiO_3_ (space group $R\bar{3}$), there are 10 atoms in the primitive cell. All the 30 modes (including 3 acoustic modes) are composed of 4 irreducible representations (irreps).

5 $$\Gamma (R\bar{3})=5{{\rm{A}}}_{{\rm{g}}}\oplus 10{{\rm{E}}}_{{\rm{g}}}^{\ast }\oplus 5{{\rm{A}}}_{{\rm{u}}}\oplus 10{{\rm{E}}}_{{\rm{u}}}^{\ast }$$

In these modes, the three acoustic modes are composed of one A_u_ and two E_u_^*^ modes. In the optical modes, A_g_ and E_g_^*^ are Raman active, while A_u_ and E_u_ are infrared (IR) active. The corresponding zone-center optical phonon modes are presented in Table 3. In its perovskite-type paraelectric phase (space group Pnma), there are 20 atoms in the primitive cell. All the 60 phonon modes at Г point (including three acoustic modes) are composed of 8 irreps.

6 $$\Gamma ({\rm{Pnma}})=7{{\rm{A}}}_{{\rm{g}}}\oplus 5{{\rm{B}}}_{1{\rm{g}}}\oplus 7{{\rm{B}}}_{2{\rm{g}}}\oplus 5{{\rm{B}}}_{3{\rm{g}}}\oplus 8{{\rm{A}}}_{{\rm{u}}}\oplus 10{{\rm{B}}}_{1{\rm{u}}}\oplus 8{{\rm{B}}}_{2{\rm{u}}}\oplus 10{{\rm{B}}}_{3{\rm{u}}}$$

**Table 3:** The calculated frequency (cm^−1^) of zone-center optical phonon modes of ilmenite (IL)-type ZnTiO_3_ (space group $R\overline{3}$).

A_u_	A_g_	E_u_	E_g_
182.88	137.31	187.49	172.99
368.25	213.20	267.39	248.74
469.14	356.79	387.31	309.73
660.88	442.07	481.90	453.06
	678.17		578.94

**Figure 6:**
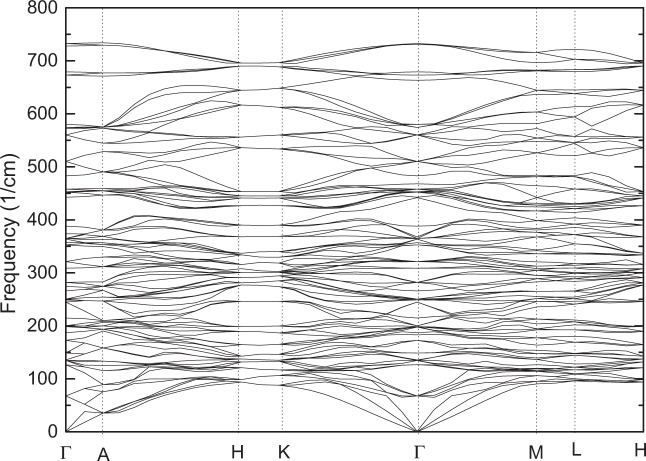
The phonon dispersion relations of the ilmenite (IL)-type ZnTiO_3_ (space group $R\overline{3}$) along high symmetry directions based on GGA.

**Figure 7:**
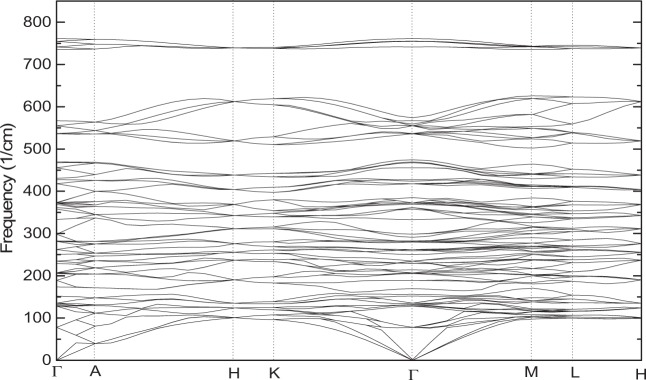
The phonon dispersion relations of the LN-type ZnTiO_3_ (space group R3c) along high symmetry directions based on GGA.

Among them, three acoustic modes are in B_1u_, B_2u_ and B_3u_, respectively. The optical modes of A_g_, B_1g_, B_2g_ and B_2g_ are Raman active; the optical modes of B_1u_, B_1u_ and B_3u_ are IR active, while the A_u_ modes are silent. We present the corresponding zone-center optical phonon modes in Table 4. From this table, it is found that there are two imaginary frequencies in B_2u_ (124.12i) and B_3u_ (101.57i), respectively. However, in its high-symmetry IL-type ZnTiO_3_, according to Table 3, it is found that there are no imaginary frequencies in the zone-center phonon optical modes; additionally, we also calculated its phonon dispersion spectrum, and it is found that there are no imaginary frequencies in these phonon modes. In order to verify the stability of the R3c and R-3 structures, we also carried out the phonon dispersion relationship based on GGA. Figures 6 and 7 present the phonon dispersion relations of the IL-type (space group $R\bar{3}$) and LN-type ZnTiO_3_ (space group R3c) along high symmetry directions. According to these two figures, it can be clearly seen that phonon frequencies of the two structures at the Г point are both zero. The calculated total energies based on norm-conserving pseudopotentials in its paraelectric (IL-type $R\bar{3}$ and Pv-type pnma) and ferroelectric phases (LN-type R3c) are −8308.8749 ($R\bar{3}$), −8308.37359 (pnma) and −8308.5650 (R3c) eV within LDA; and −8321.4057 ($R\bar{3}$), −8320.7809 (pnma) and −8321.0790 (R3c) eV within GGA. The results show that the energy of its IL-type paraelectric phase is the lowest, the energy of Pv-type paraelectric phase is the highest, and the energy of LN-type ferroelectric phase lies in the middle. In order to verify whether it is due to the inaccuracy of the exchange correlation, we have carried out total energy calculation based on PBEsol, which is intended to improve on PBE for equilibrium properties such as bond lengths and lattice parameters. The calculated lattice parameters of PBEsol are also presented in Table 1. The results show that total energies are −8302.8974 ($R\bar{3}$), −8302.3911 (pnma) and −8302.5810 (R3c) eV based on PBEsol. This is consistent with the above conclusion E($R\bar{3}$) < E(R3c) < E(pnma). This should be attributed to the synthesis of LN-type ZnTiO3 under high pressure.

**Table 4:** The frequencies (cm^−1^) of zone-center optical phonon modes in the perovskite (Pv)-type ZnTiO_3_.

A_g_	A_u_	B_1g_	B_2g_	B_3g_	B_1u_	B_2u_	B_3u_
105.09	74.20	173.82	137.82	131.12	63.56	124.12i	101.57i
153.48	102.07	244.52	163.07	304.52	111.09	74.88	95.45
241.88	113.28	356.88	250.55	454.52	183.91	127.15	178.83
300.53	151.47	467.27	370.45	462.60	305.34	219.98	223.22
434.00	283.65	654.80	467.71	747.05	332.94	397.35	325.38
451.84	379.67		509.34		370.06	413.30	366.11
509.22	394.47		729.64		440.83	472.24	430.89
	490.47				461.78		489.77
					516.84		547.12

In the ferroelectric phase (space group R3c) in ZnTiO_3_, the primitive cell contains 10 atoms, so there are 30 phonon modes at the Г point. The three zero-frequency acoustic modes comprise one in A_1_ and two in E. The zone-center phonon optical modes are as follows:

7 $$\Gamma ({\rm{R}}3{\rm{c}})=4{{\rm{A}}}_{1}\oplus 5{{\rm{A}}}_{2}\oplus 9{\rm{E}}$$

In these modes, A_1_ and E optical modes are both Raman and infrared (IR) active, while A_2_ modes are silent. The optical modes of the LN-type ZnTiO_3_ (see in Table 5) infer that the imaginary frequencies disappear from perovskite-type paraelectric phase to LiNO_3_-type ferroelectric phase. Due to A1 and E optical modes being IR active, these frequencies are splitting into LO and TO modes described by the Lyddane–Sachs–Teller (LST) relation^50^. Generally, the large Bern effective charge corresponds to a large LO-TO splitting. From Table 5, one can see that three A_1_ modes (281.53~325.70 cm^−1^, 387.55 ~436.30 cm^−1^, and 653.93~826.10 cm^−1^) and three E modes (343.16~428.20 cm^−1^, 436.96~479.11 cm^−1^, and 657.30~848.01 cm^−1^) show large LO-TO splitting. In Table 6, we present the mode effective charges and mode-oscillator strengths corresponding to the zone-center transverse optical (TO) modes of LN-type ZnTiO_3_.

**Table 5:** The frequencies (cm^−1^) of transverse optical (TO) and longitudinal optical (LO) zone-center optical phonon modes of the LiNbO_3_-type ZnTiO_3_.

A_1_ (TO)	A_1_ (LO)	E (TO)	E (LO)	A2
142.56	165.26	141.16	152.71	120.69
281.53	325.70	194.64	204.87	363.21
387.55	436.30	223.63	223.88	417.04
653.83	826.10	265.30	267.41	468.54
		321.53	325.71	839.51
		343.16	428.20	
		436.96	479.11	
		639.91	656.14	
		657.30	848.01

**Table 6:** The calculated mode effective charges (|e|), and mode-oscillator strengths (within 10^−4^ atomic units) corresponding to the zone-center transverse optical phonon modes of LiNbO_3_-type ZnTiO_3_.

A_1_ modes (ZnTiO_3_)			E modes (ZnTiO_3_)		
ω	Z_m_^*^	S_m_	ω	Z_m_^*^	S_m_
142.56	3.61	1.97	141.16	2.47	1.09
281.53	6.51	10.05	194.64	2.50	1.01
387.55	0.50	0.08	223.63	0.30	0.02
653.83	5.63	10.35	265.30	1.18	0.40
			321.53	3.68	2.54
			343.16	6.02	10.07
			436.96	1.33	0.59
			639.91	6.15	12.06
			657.30	1.73	0.84

### Piezoelectric properties

In order to investigate the structural stability and piezoelectric properties of LN-type ZnTiO_3_, we calculate the elastic and piezoelectric constants by treating homogeneous strains as perturbations based on DFPT. According to the calculated results, the six independent elastic coefficients (Voigt notations) of this compound are C_11_, C_12_, C_13_, C_14_, C_33_, and C_44,_ respectively. Additionally, it should be pointed out that C_66_ = (C_11_ − C_12_)/2. In Table 7, we give the corresponding elastic stiffness coefficients (relaxed ion) of LN-type ZnTiO_3_. The point group of LN-type ZnTiO_3_ belongs to the triangle crystal. As discussed in refs. ^51,52^, the triangle crystals need to satisfy the following Born mechanical stability criterion.

8 $$\begin{array}{c}{C}_{11}-|{C}_{12}| > 0\\ ({C}_{11}+{C}_{12}){C}_{33}-2{C}_{13}^{2} > 0\\ ({C}_{11}-{C}_{12}){C}_{44}-2{C}_{15}^{2} > 0\end{array}$$

**Table 7:** The Elastic stiffness coefficients of LN-type ZnTiO_3_.

Elastic stiffness coefficients C_ij_ (10^11^N/m^2^)							
material	C_11_	C_12_	C_13_	C_15_	C_33_	C_44_	C_66_
ZnTiO_3_	3.675	1.875	1.442	0.031	2.877	0.800	0.899
ZnSnO_3_ ref. ^43^	1.94	0.63	1.01	0.001	1.69	1.17	0.92
	C_11_	C_12_	C_13_	C_14_	C_33_	C_44_	C_66_
LiNbO_3_ ref. ^44^	2.03	0.53	0.75	0.09	2.45	0.60	0.75
LiTaO_3_ ref. ^44^	2.298	0.440	0.812	−0.104	2.798	0.968	0.929

The results presented in Table 7 reveal that the elastic constants of the LN-type ZnTiO_3_ satisfy the constraints of the Born stability condition. Therefore, the structure of LN-type ZnTiO_3_ is stable. By using homogeneous strains and electric fields as perturbations, we can calculate the piezoelectric properties of LN-type ZnTiO_3_. The obtained piezoelectric tensor of this compound has the four independent elements (Voigt notations) e_11_, e_15_, e_31_, e_33_:

9 $$e=(\begin{array}{cccccc}{e}_{11} & -{e}_{11} & 0 & 0 & {e}_{15} & 0\\ 0 & 0 & 0 & {e}_{15} & 0 & 0\\ {e}_{31} & {e}_{31} & {e}_{33} & 0 & 0 & 0\end{array})$$

Obviously, this tensor is the same as that of LN-type ZnSnO_3_ and ZnGeO_3_, however it is a little different from that of LiNbO_3_ and LiTaO_3_ since independent elements of the piezoelectric tensor in LiNbO_3_ and LiTaO_3_ are e_22_, e_15_, e_31_, and e_33_. In Table 8, we present the piezoelectric constants of LN-type ZnTiO_3_, ZnSnO_3_ and ZnGeO_3_ together with LiNbO_3_ and LiTaO_3_^30,53^. From this table, the piezoelectric constants e_11_, e_15_, e_31_, e_33_ are −0.93, 1.00, 1.01 and 2.51 C/m^2^, respectively. It is well known that lead titanate (PbTiO_3_) piezoelectric ceramics have excellent piezoelectric properties^54,55^. By comparing the piezoelectric coefficients of LN-type ZnTiO and that of PbTiO_3_^55^ calculated by density functional pertubation theory and finite strain method (see Table 8), it is found that values of e_31_, e_33_ of LN-type ZnTiO_3_ are close to and exceeds one-half of that of PbTiO_3,_ and the value of e_15_ is relatively small compared to that of PbTiO_3_. The large piezoelectric constants reveal that LN-type ZnTiO_3_ is a promising candidate piezoelectric material.

**Table 8:** The piezoelectric stress constants (relaxed ion) of LN-type ZnTiO_3_, it is noted that a and b in PbTiO_3_ represents DFPT finite strain method, respectively.

Piezoelectric stress constants e_ij_ (C/m^2^)				
Material	e_11_	e_15_	e_31_	e_33_
ZnTiO_3_	−0.93	1.00	1.01	2.51
PbTiO_3_ ref. ^54^ ^a^		6.63	2.06	4.41
PbTiO_3_ ref. ^54^ ^b^		6.66	2.07	4.48
ZnSnO_3_ ref. ^43^	−0.15	0.26	1.23	0.29
ZnGeO_3_ ref. ^43^	−0.27	0.36	0.65	2.81
	**e**_**22**_	**e**_**15**_	**e**_**31**_	**e**_**33**_
LiNbO_3_ ref. ^44^	2.43	3.76	0.23	1.33
LiTaO_3_ ref. ^44^	1.67	2.72	−0.38	1.09

### Nonlinear optical properties

As mentioned above, the zone-center phonon optical modes of ferroelectric phase ZnTiO_3_ can be divided into 4A_1_ ⊕ 5A_2_ ⊕ 9E, and A_1_ and E modes are both Raman and infrared active. The Raman scattering efficiencies of the phonon modes can be calculated using the following formula^56,57^:

10 $$\frac{dS}{d\Omega }=\frac{{({\omega }_{0}-{\omega }_{m})}^{4}}{{c}^{4}}{|{{\rm{e}}}_{{\rm{s}}}\cdot {\alpha }_{m}\cdot {{\rm{e}}}_{0}|}^{2}\frac{\hslash }{2{\omega }_{m}}({n}_{m}+1)$$

where *α*_*m*_, c, $\hslash$ and *n*_*m*_ are the Raman susceptibility, the speed of light in vacuum, the Planck constant and Bose-Einstein factor, respectively; *ω*_0_ and *ω*_*m*_ are the frequencies of an incoming photon and the mth zone-center phonon mode, the frequency of scattered outgoing photon is (*ω*_0_ − *ω*_*m*_), e_s_ and e_0_ indicate incoming and outgoing polarizations within an angle Ω. According to the structural symmetry, the Raman tensors of A_1_ mode (along z axis) and E mode (in the x-y plane) of LN-type ZnTiO_3_ are similar to that of LiNbO_3_ and described as follows:

11 $${{\rm{A}}}_{1}({\rm{z}})=(\begin{array}{ccc}{\rm{a}} & 0 & 0\\ 0 & {\rm{a}} & 0\\ 0 & 0 & {\rm{b}}\end{array})$$

12 $$E(x)=(\begin{array}{ccc}{\rm{c}} & {\rm{0}} & {\rm{d}}\\ {\rm{0}} & -{\rm{c}} & {\rm{0}}\\ {\rm{d}} & {\rm{0}} & {\rm{0}}\end{array})\,E(y)=(\begin{array}{ccc}{\rm{0}} & {\rm{c}} & {\rm{0}}\\ {\rm{c}} & {\rm{0}} & -{\rm{d}}\\ {\rm{0}} & -{\rm{d}} & {\rm{0}}\end{array})$$

**Figure 8:**
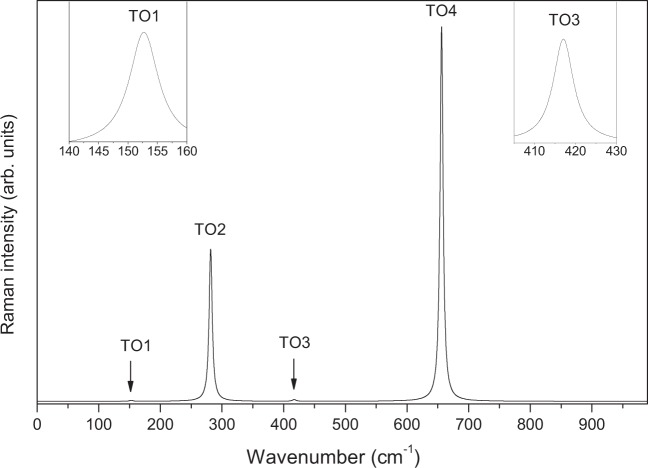
The calculated Raman spectra of LiNbO_3_-type ZnTiO_3_ for a x(zz)y scattering configuration in the range of 0~1000 cm^−1^. The spectra displays the TO of A_1_ modes.

**Figure 9:**
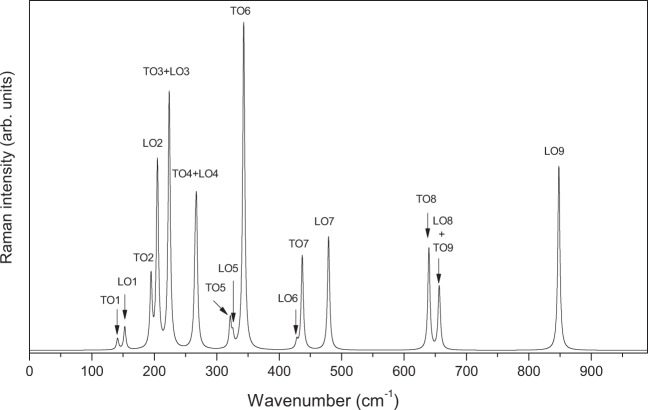
The calculated Raman spectra of the LiNbO_3_-type ZnTiO_3_ for a x(yz)y scattering configuration of E modes in the range of 0~1000 cm^−1^.

Figure 8 displays the calculated Raman spectrum of LiNbO_3_-type ZnTiO_3_ for the x(zz)y scattering configuration. In this configuration, as in the case of LiNbO_3_ or PbTiO_3_, only the transverse A_1_ modes can be detected. In order to see the peaks of TO1 and TO3 clearly, we have made two enlarged small illustrations in this figure. The results show that TO2 and TO4 modes have a very strong Raman scattering efficiency. In comparison, the Raman scattering efficiencies of TO1 and TO3 are very weak and are much smaller than that of the TO2 and TO4 modes. As presented in Tables 5 and Table 6, notice that TO2 and TO4 of A_1_ modes have large LO-TO splittings and oscillator strengths, and the two frequencies just correspond to large Raman peaks. Figure 9 presents the calculated Raman spectrum of LiNbO_3_-type ZnTiO_3_ for the x(yz)y configuration. In this configuration, the same as LiNbO_3_, only the TO and LO of E modes can be detected. From this figure, it is found that five transverse modes (TO3, TO4, TO6 and TO7 and TO8) and five longitudinal modes (LO2, LO3, LO4, LO7 and LO9) have strong Raman scattering efficiency. Due to the LO-TO splittings of LO3(223.63 cm^−1^)-TO3(223.88 cm^−1^) and LO4(265.30 cm^−1^)-TO4(267.41 cm^−1^) being very small, the two Raman intensity peaks almost completely overlap.

The LN-type ZnTiO_3_ belongs to the 3 m point group, therefore this compound is a candidate promising nonlinear optical material. As discussed by Inaguma *et al*.^14^, the SHG response of LN-type ZnTiO_3_ is 24 times greater than that of LN-type ZnSnO_3_ and has about 5% of the SHG response of LiNbO_3_. By using electric fields and atomic displacements as perturbations based on DFPT, we have calculated the nonlinear optical (NLO) susceptibilities and electro-optic (EO) coefficients of LN-type ZnTiO_3_. According to reports by M. Veithen, *et al*.^48^, the NLO properties of various semiconductors, such as LiNbO_3_, PbTiO3, and AlAs, etc. were calculated by DFPT based on the ABINIT package. The comparation between the theoretical results of NLO and EO coefficients with the experimental data shows that the method is reliable. The calculated results show that the three independent elements of the NLO tensor of this compound are d_12_, d_15_ and d_33_ (Voigt notations).

13 $$d=(\begin{array}{cccccc}-{d}_{12} & {d}_{12} & 0 & 0 & {d}_{15} & 0\\ 0 & 0 & 0 & {d}_{15} & 0 & {d}_{12}\\ {d}_{15} & {d}_{15} & {d}_{33} & 0 & 0 & 0\end{array})$$

The NLO tensor form of LN-type ZnTiO_3_ is the same as that of LN-type ZnSnO_3_ and ZnGeO_3_, but it is not the same as that of LiNbO_3_ and LiTaO_3_ since the independent elements of NLO tensor of the latter are d_22_, d_31_, d_33_. The calculated NLO susceptibilities of LN-type ZnTiO_3_ together with those of LN-type ZnGeO_3_^43^, LN-type ZnSnO_3_^31^, LiNbO_3_^58^ and LiTaO_3_^59^ are presented in Table 9. From this table, the NLO susceptibilities d_12_, d_15_ and d_33_ are 1.37, 1.46 and −20.18 Pm/V, respectively, and one can see that the biggest susceptibility (d_33_) of this compound is larger than that of ZnSnO_3_, and lower than that of LiNbO_3_. For the electro-optic (EO) coefficients of LN-type ZnTiO_3_, the results reveal that its independent elements are γ_11_, γ_13_, γ_33_, and γ_51_.

14 $$\gamma =(\begin{array}{ccc}{\gamma }_{11} & 0 & {\gamma }_{13}\\ -{\gamma }_{11} & 0 & {\gamma }_{13}\\ 0 & 0 & {\gamma }_{33}\\ 0 & {\gamma }_{51} & 0\\ {\gamma }_{51} & 0 & 0\\ 0 & -{\gamma }_{11} & 0\end{array})$$

**Table 9:** The calculated nonlinear optical susceptibilities of LN-type ZnTiO_3_.

Nonlinear optical susceptibilities d_ij_ (Pm/V)			
	d_12_	d_31_	d_33_
ZnTiO_3_	1.37	1.46	−20.18
ZnSnO_3_ ref. ^31^	13.09	1.73	11.06
ZnGeO_3_ ref. ^30^	3.14	6.16	8.45
	**d**_**22**_	**d**_**31**_	**d**_**33**_
LiNbO_3_ ref. ^56^	−6.25	3.60	−37.5
LiTaO_3_ ref. ^57^	−1.07	−1.07	−16.40

As can be seen, the EO tensor is the same as that of LN-type ZnSnO_3_ and ZnGeO_3,_ but is different from that of LiNbO_3_ and LiTaO_3_ since the independent elements of latter are γ_13_, γ_33_, γ_22_ and γ_51_. The EO coefficients can be divided into three contributing parts: electronic, ionic and piezoelectric. In Table 10, we give the calculated EO coefficients of LN-type ZnTiO_3_. For ease of comparison, the EO coefficients of LN-type ZnGeO_3_, LN-type ZnSnO_3_, LiNbO_3_^60^ and LiTaO_3_^61^ are also listed in this table. Our obtained EO coefficients γ_11_, γ_13_, γ_33_, and γ_51_ for LN-type ZnTiO_3_ are 0.46, 3.71, 17.17 and 1.62 Pm/V, respectively. Obviously, the coefficients γ_13_ and γ_33_ are much larger than that of LN-type ZnSnO_3_ and ZnGeO_3_. The large NLO susceptibilities and EO coefficients reveal that the LN-type ZnTiO_3_ is a high performance lead-free nonlinear optical crystal.

**Table 10:** The calculated Electro-optic tensor of LN-type ZnTiO_3_.

Electro-optic tensors γ_ij_ (Pm/V)				
	γ_11_	γ_13_	γ_33_	γ_51_
ZnTiO_3_	0.46	3.71	17.17	1.62
ZnSnO_3_ ref. ^30^	1.23	1.57	−2.78	0.54
ZnGeO_3_ ref. ^31^	0.44	1.68	5.56	0.85
	**γ**_**22**_	**γ**_**31**_	**γ**_**33**_	**γ**_**33**_
LiNbO_3_ ref. ^58^	8.6	30.8	3.4	28
LiTaO_3_ ref. ^59^	35	8.2	20	0.5

## Conclusions

In this work, the electronic structure, zone-center phonon modes, piezoelectric and nonlinear optical properties of LN-type ZnTiO_3_ are investigated by first-principles calculations based on DFT. The electronic structure shows that this compound is a wide direct-band-gap insulator. By investigating the zone-center phonon modes of the paraelectric and ferroelectric phases, it is found that there are two imaginary frequencies in B_2u_ and B_3u_ in the perovskite paraelectric phase, and subsequently the imaginary frequencies disappear in the ferroelectric phase. The calculated spontaneous polarizations are 90.43 μC/cm^2^ and 93.14 μC/cm^2^ based on LDA and GGA, respectively. Our results are in good agreement with the experimental results, and the large spontaneous polarization reveals that this compound is a good ferroelectric material.

The elastic constants of LN-type ZnTiO_3_ satisfy the constraints of the Born stability condition, and therefore this compound has a stable structure. The obtained piezoelectric tensor has four independent elements e_11_, e_15_, e_31_, e_33,_ with values −0.93, 1.00, 1.01 and 2.51 C/m^2^. This shows that this compound is a promising piezoelectric crystal. Like LiNbO_3_, the calculated Raman spectrum for the x(zz)y and x(yz)y configurations correspond to A_1_ and E modes, and the Raman scattering peaks of A_1_ and E modes are assigned to their zone-center optical modes, respectively. The independent nonlinear optical susceptibilities of this compound are d_12_, d_15_ and d_33_ with values 1.37, 1.46 and −20.18 Pm/V, respectively. For the EO coefficients, the independent elements are γ_11_, γ_13_, γ_33_, and γ_51_ with values 0.46, 3.71, 17.17 and 1.62 Pm/V, respectively. The results show that LN-type ZnTiO_3_ exhibits better nonlinear optical properties than LN-type ZnSnO_3_. The large piezoelectric and nonlinear optical susceptibilities reveal that this compound is a high-performance lead-free piezoelectric and nonlinear optical crystal.
